# Sex Differences in Antiretroviral Therapy Initiation in Pediatric HIV Infection

**DOI:** 10.1371/journal.pone.0131591

**Published:** 2015-07-07

**Authors:** Masahiko Mori, Emily Adland, Paolo Paioni, Alice Swordy, Luisa Mori, Leana Laker, Maximilian Muenchhoff, Philippa C. Matthews, Gareth Tudor-Williams, Nora Lavandier, Anriette van Zyl, Jacob Hurst, Bruce D. Walker, Thumbi Ndung’u, Andrew Prendergast, Philip Goulder, Pieter Jooste

**Affiliations:** 1 Department of Paediatrics, University of Oxford, Oxford, United Kingdom; 2 Kimberley Hospital, Kimberley, Durban, South Africa; 3 Department of Paediatrics, Imperial College, London, United Kingdom; 4 Nuffield Department of Medicine, University of Oxford, Oxford, United Kingdom; 5 Ragon Institute of MGH, MIT and Harvard, Boston, MA, United States of America; 6 Doris Duke Medical Research Institute, University of KwaZulu-Natal, Durban, South Africa; 7 KwaZulu-Natal Research Institute for Tuberculosis and HIV (K-RITH), University of KwaZulu-Natal, Durban, South Africa; 8 Max Planck Institute for Infection Biology, Berlin, Germany; 9 Centre for Paediatrics, Blizard Institute, Queen Mary University of London, London, United Kingdom; University of Cape Town, SOUTH AFRICA

## Abstract

The incidence and severity of infections in childhood is typically greater in males. The basis for these observed sex differences is not well understood, and potentially may facilitate novel approaches to reducing disease from a range of conditions. We here investigated sex differences in HIV-infected children in relation to antiretroviral therapy (ART) initiation and post-treatment outcome. In a South African cohort of 2,101 HIV-infected children, we observed that absolute CD4+ count and CD4% were significantly higher in ART-naïve female, compared to age-matched male, HIV-infected children. Absolute CD4 count and CD4% were also significantly higher in HIV-uninfected female versus male neonates. We next showed that significantly more male than female children were initiated on ART (47% female); and children not meeting criteria to start ART by >5yrs were more frequently female (59%; p<0.001). Among ART-treated children, immune reconstitution of CD4 T-cells was more rapid and more complete in female children, even after adjustment for pre-ART absolute CD4 count or CD4% (p=0.011, p=0.030, respectively). However, while ART was initiated as a result of meeting CD4 criteria less often in females (45%), ART initiation as a result of clinical disease in children whose CD4 counts were above treatment thresholds occurred more often in females (57%, p<0.001). The main sex difference in morbidity observed in children initiating ART above CD4 thresholds, above that of TB disease, was as a result of wasting and stunting observed in females with above-threshold CD4 counts (p=0.002). These findings suggest the possibility that optimal treatment of HIV-infected children might incorporate differential CD4 treatment thresholds for ART initiation according to sex.

## Introduction

Sex differences in susceptibility and mortality from infectious diseases in childhood contribute to the greater burden of disease and death observed in males throughout life [1]. In childhood infections these sex differences are perhaps most consistent amongst parasitic infections [2], but also exist across the majority of viral and bacterial infections [1]. However, the mechanisms underlying these sex differences in childhood infections are poorly understood. As sex differences in the important non-specific effects and overall outcome of vaccines are better recognized [3–6], identifying sex disparities in childhood infections and, ultimately, understanding the basis for them, is also becoming increasingly important.

We here focus on sex differences in pediatric HIV infection and specifically on the initiation of antiretroviral therapy (ART) and post-treatment outcome in HIV-infected children. Although prevention of mother-to-child transmission (PMTCT) programs have dramatically reduced new pediatric infections over the past decade, coincidentally the increasing success of ART programs has meant that the number of children living with HIV, now estimated at 3.2 million (http://www.avert.org/children-and-hiv-aids.htm), has continued to grow.

The optimal timing of ART initiation in pediatric HIV infection [7] is an increasingly important topic in the face of the more widespread access to therapy. The World Health Organization (WHO) guidelines for ART initiation in children (http://www.who.int/hiv/pub/guidelines/arv2013) have, over time, favored increasingly early treatment. This suggests that numbers of ART-treated older children will grow further. There is also a sizeable epidemic of adolescents with HIV who have survived in the absence of ART [8]. Together these data suggest that the pediatric HIV epidemic is one that is changing: growing in size, comprising a rising number of children on ART and giving rise to increasing proportion of older children and adolescents.

In the absence of ART children progress more rapidly to disease than infected adults [9], with approximately 50% developing AIDS by one year, and >50% dying by two years in sub-Saharan Africa [9–11]. This compares with a median time to AIDS of approximately 10 years in untreated adult HIV infection [12]. ART guidelines differ in children, both because of this increased risk of disease progression, and because absolute CD4 counts change with age through normal childhood [13]. From 2008 until 2013, WHO proposed ART initiation in all HIV-infected infants (children aged <1 year old), irrespective of CD4 count; in children 1–4yrs with a CD4+ T cell percentage (CD4%) <25% or absolute CD4 count <750/ul; and in children ≥5yrs with absolute CD4 counts <350/ul. Since July 2013, WHO guidelines recommend ART initiation in all HIV-infected children aged 5yr or younger, and in HIV-infected children >5yr with absolute CD4 counts <500/ul. In addition to age and CD4 thresholds, children meeting clinical criteria (WHO clinical disease stage 3 or 4) are recommended for ART.

Data describing the impact of sex differences in ART initiation and post-treatment outcome in HIV-infected children in sub-Saharan Africa are relatively sparse. In adult HIV infection, viral loads (VLs) are approximately 0.5 log copies/ml lower [14–17], and absolute CD4 counts 100/ul higher in women compared to men [18,19], but progression to HIV disease occurs at the same rate and ART initiation guidelines have not differed between men and women [16]. More recently, higher CD4 T cell counts and lower VLs have been reported in HIV-infected female children and higher CD4 counts also in HIV-exposed, uninfected female children [20, 21]. No differences between males and females in HIV mortality have been reported in infected children followed from birth in the pre-ART era [11]. However, higher mortality was observed in two studies in female children treated with ART [21, 22], prompting the hypothesis that ART is initiated too late in HIV-infected female children due to intrinsically higher CD4 counts pre- and post-infection. However, this finding of increased mortality in female children post ART initiation has not been described in other studies [23–25].

We here investigate the impact of sex differences in ART initiation and post-treatment outcome in >2,500 HIV-infected and uninfected children in South Africa.

## Materials and Methods

### Subjects and data collection

Data were analyzed from 2,452 South African children, comprising 2,101 HIV-infected children (0–13 yo) attending Kimberley Hospital outpatient clinic and 351 HIV-exposed uninfected (HEU) neonates (born to HIV-infected mothers) followed in Durban, South Africa [26, 27] (S1 Table). The Kimberley cohort comprised children who were tested and diagnosed either following presentation with HIV disease, or as a result of presentation of a relative (mother or sibling) with a new diagnosis of HIV disease. These children were not followed from birth therefore. The Durban cohort of children followed from for the first month from birth was part of a research study designed to identify in utero and intra partum HIV infection, more fully described elsewhere [26, 27], in the course of which expose-uninfected children were also followed for this period of time. The Kimberley data analyzed extended from July 2003 to March 2013 and comprised date of birth, sex, date of ART initiation, and 6-monthly CD4+ T-cell counts, CD4%, and VL. All HIV-infected children attending the Kimberley Hospital outpatient clinic were included in the analyses; there were no exclusion criteria other than these two (ie HIV infection, and attending the Kimberley Hospital outpatient clinic). Analyses were undertaken on these study subjects according to availability of data, depending on follow up in Kimberley or not, and the presence of absolute CD4 count, CD4% and viral load data or not.

VLs up to 2010 were measured using the BioMérieux NucliSens Version 2.0 assay (range 20–10,000,000 copies/ml) and thereafter using the Roche COBAS AmpliPrep/COBAS TaqMan HIV-1 Test v2.0 (range 20–10,000,000 copies/ml).

First-line ART regimens in the Kimberley cohort were, in 2003, for children aged >3yrs or >13kg, stavudine, lamivudine, and efavirenz; and for children aged <3 yo or who were <13kg, stavudine, lamivudine and nevirapine. In 2004, lopinavir/ritonavir (Kaletra) replaced nevirapine in first-line regimens; and in 2010, abacavir replaced stavudine. In 2013 the first line regimen for children >40kg was a combination of tenofovir, emtricitabine, and efavirenz. ART was initiated as per South African guidelines (http://www.sahivsoc.org/practise-guidelines/national-dept-of-health-guidelines#) with changes over time as described above.

This research was approved by the ethical review boards at each site, the University of KwaZulu-Natal, South Africa; the University of the Free State, South Africa; and the University of Oxford, UK. Next of kin, or guardians gave their informed, written consent, that was documented and retained on the Study Consent Forms, on behalf of the minors/children enrolled in the study, in accordance with the protocols approved by above-listed ethics committees.

### Statistical Analysis

Associations between sex differences or age and clinical outcome were analyzed using Excel 2007 and SPSS 21.0. In treatment-naïve children and HEU infants, sex differences in CD4 counts, CD4%, and VL were compared using the Mann-Whitney U-test, and a linear regression model for multivariate analysis. In children receiving ART, sex differences in outcome were analyzed by the log rank test and Cox proportional hazards models.

## Results

### Study cohorts and subgroups analyzed

**Fig 1:**
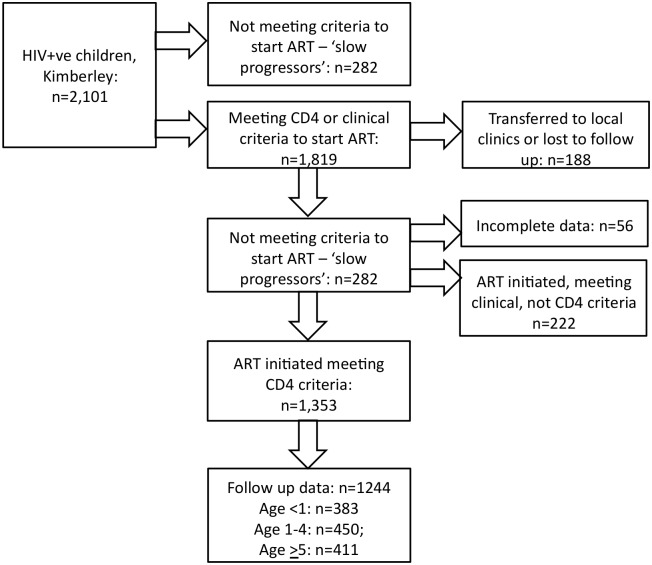
Study cohorts of HIV-infected South African children analyzed.

**Fig 2:**
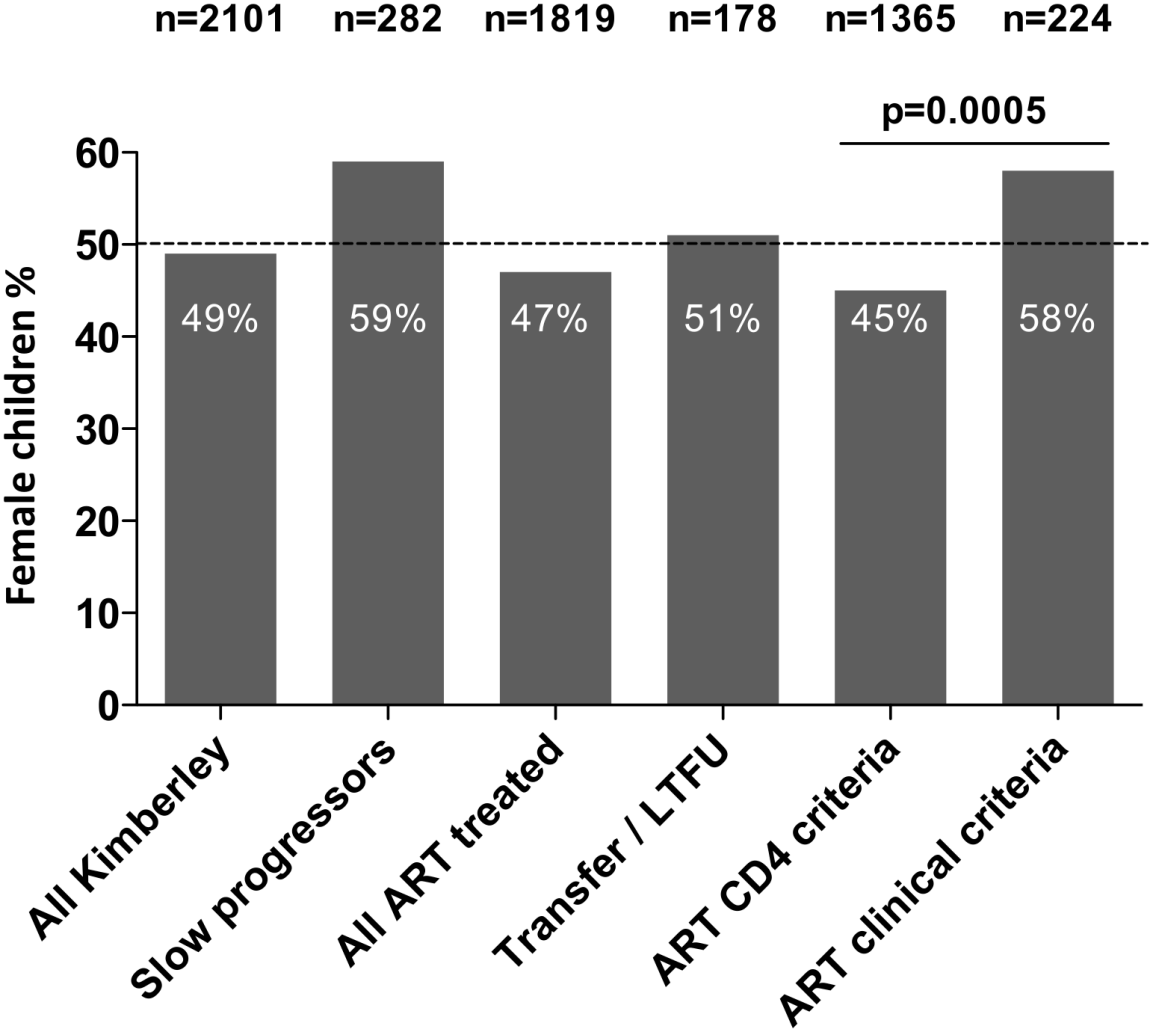
Proportion of HIV-infected children who were female in the subgroups analyzed within the Kimberley cohort. The proportion of female children in whom ART was initiated at CD4 counts higher than the CD4 thresholds compared to those in whom ART was initiated at CD4 counts lower than the CD4 thresholds differed significantly (p<0.001).

The Kimberley study cohorts are represented in Fig 1. Of 2,101 HIV-infected children in total attending the Kimberley hospital outpatient clinic, ART was initiated in 1,819; of these, post-ART CD4 data were unavailable in 188 subjects (52% female), either because of care being transferred to other clinics or loss to follow up. Of the remaining 1,631 subjects, CD4 data were incomplete in 56 cases; in 222 cases ART was initiated because of meeting clinical criteria, as opposed to CD4 criteria. Post-treatment follow up data (all of CD4+ T cell count, CD4%, viral load, and survival) were available for 1,244 of the 1,353 children in whom ART was initiated. The proportion of children who were female in the subgroups described is shown in Fig 2.

### ART-naïve HIV-infected females have higher absolute CD4 counts than males

**Fig 3:**
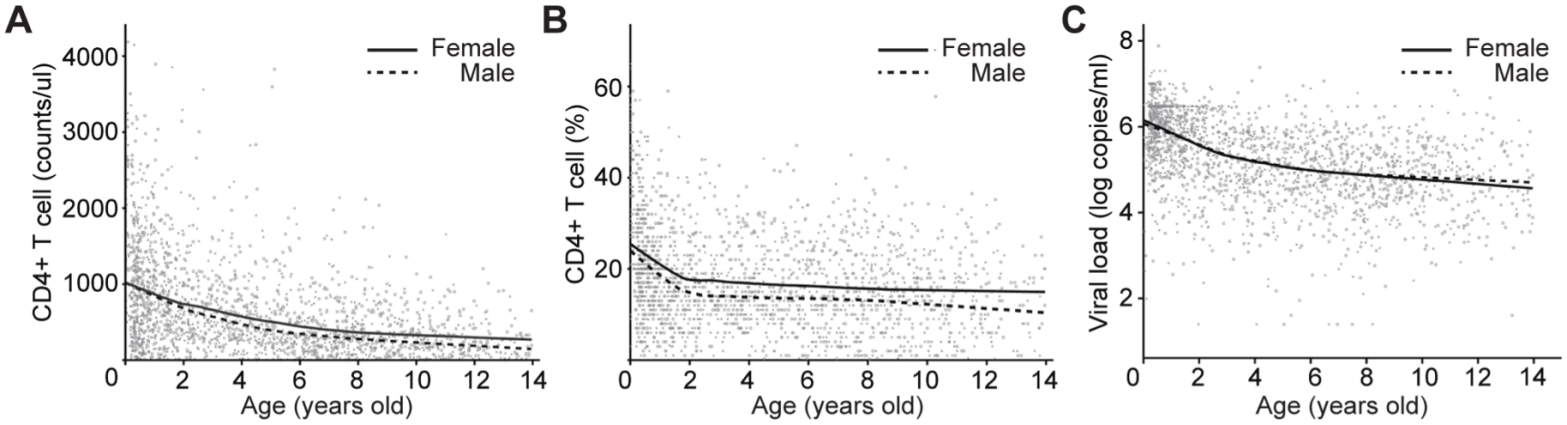
Sex differences in CD4+ T cell count, CD4% and viral load, amongst 2,101 ART-naïve South African children. A. Absolute CD4 counts changes with age. B. CD4% changes with age. C. Viral load changes with age. In each panel, the solid lines are Loess-smoothed regression lines for female children and the dotted lines are Loess-smoothed regression lines for male children. A multivariable linear regression model, with both sex and age as covariates, shows significantly lower absolute CD4 counts in males (p = 0.005); significantly lower CD4% in males (p = 3.7x10^-7^); and no significant difference in viral load between the sexes.

Initial analyses were undertaken of all 2,101 HIV-infected children enrolled, using the immediate pre-ART timepoint in those (n = 1819) who received ART, and the enrollment timepoint for the slow progressor children (n = 282) who never received ART. Among these ART-naïve, HIV-infected children studied, absolute CD4 counts and CD4% were significantly higher in females (median 481/ul vs 444/ul, p = 0.013; 17% vs 14%, p<0.001, Table 1, Fig 3). CD4 counts were a mean of 88/ul higher and CD4% a mean of 3% higher in females compared to males aged 0–13yrs. Although there was no sex difference in viral load in the cohort overall pre-ART, males aged ≥12yr showed a marginally higher viral load compared to females (4.7 vs 4.5 log c/ml, p<0.001, Mann-Whitney U-test).

**Table 1:** Absolute CD4 count, CD4% and viral load in HIV-infected children and HIV-uninfected neonates. HIV-Infected Children, n = 2,101.

	**Female**				**Male**				**p value** ^a^
		Median	IQR			Median	IQR		
**Age (yrs)**	n = 1022	4yrs	1–7		n = 1079	3yrs	1–7		0.10
**CD4 (/ul)**	n = 1005	481	241–917		n = 1061	444	211–866		0.013
**CD4%**	n = 910	17	11–25		n = 954	14	9–21		<0.001
**VL (log)**	n = 946	5.3	4.6–5.9		n = 1027	5.3	4.7–5.9		0.27
	**Age:**	**years**							
		**<1**	**1**	**2**	**3**	**4–5**	**6–7**	**8–10**	**11–13**
n = 2,101		480	248	175	151	269	294	334	150
**CD4 (/ul)**	**f**	843	668	723	634	464	351	313	255
	**m**	887	731	574	440	395	269	250	152
**CD4%**	**f**	20	17	16	15	15	16	15	17
	**m**	19	14	14	13	13	12	14	10
**VL (log)**	**f**	6.04	5.80	5.36	5.20	5.05	4.89	4.76	4.57
	**m**	6.00	5.73	5.49	5.22	5.08	4.89	4.86	4.81
HIV- uninfected infants,n = 351(180 females; 171 males)									
		**Age:**	**day 1**	**IQR**	**p value** ^**a**^		**4 wks**	**IQR**	**p value** ^**a**^
	**Age (day)**	**f**	1	1–2			27	27–29	
		**m**	1	1–2	0.26		28	27–29	0.11
	**CD4 (/ul)**	**f**	1664	1267–2210			2540	2030–3064	
		**m**	1398	1059–1896	<0.0001		2324	1837–2779	0.005
	**CD4%**	**f**	52	44–58			45	40–50	
		**m**	50	43–56	0.11		42	35–48	<0.0001

^**a**^ Mann Whitney test

### In uninfected neonates absolute CD4 count and CD4% are higher in females

To determine whether the differences in CD4 counts and CD4% described above were independent of HIV infection, we also analyzed sex differences in CD4 counts and CD4% among 351 HIV-exposed, uninfected (HEU) neonates. Of these, 180 (51%) were female and 171 (49%) male (Table 1). Both on the first day of life, and at one month of age, absolute CD4 count and CD4% was higher in female infants (Table 1). These data indicate that sex differences in CD4 counts and in CD4% are also evident from birth in HIV-exposed uninfected neonates.

### Immune reconstitution more rapid and more complete in females on ART

Having shown that CD4 counts are higher in female than male HIV-infected children, and that these differences exist from birth, we then sought to address the question of whether ART may be initiated at a later time than would be optimal in female children, since, in the absence of clinical indications, ART initiation is principally based on CD4 counts.

**Fig 4:**
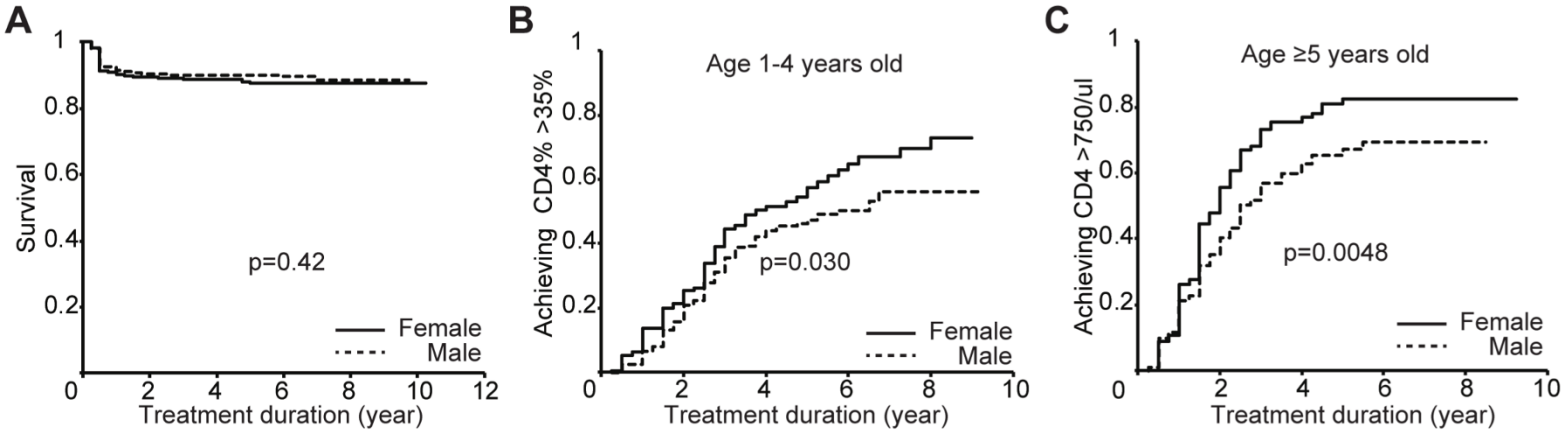
Sex differences in immune reconstitution amongst the patients started treatment under the pre-2013 WHO guidelines. Sex differences by log rank test are shown as follows: A. Survival after ART initiation. B. CD4+ T cell percentage recovery (>35%) rate among the children who started ART aged 1–4 years old with CD4+ T cell <25%. C. Absolute CD4+ T cell count recovery (>750/ul) among children initiating ART aged ≥5 years old with absolute CD4+ T cell counts <350/ul.

We first analyzed treatment outcomes among the 1,244 children in whom ART was initiated based on WHO age criteria (<1yr) or CD4 criteria (described in the Methods). There was no sex difference in survival among these children (p = 0.42, Fig 4A), nor in the time to achieve viral suppression (<50 copies/ml, p = 0.26, not shown). However, even after adjustment for pre-ART CD4 counts or CD4% (see below), CD4+ T cell reconstitution to normal levels for age-matched uninfected children (defined as: CD4% ≥35% in children aged 1–4yr, or CD4 counts >750/ul in children aged ≥5yrs [13]) was more rapid and more complete in females (Fig 4B and 4C). Prior to ART initiation, the differences in CD4% and in CD4 counts did not reach statistical significance comparing male with female children (S2 Table), and, using the Cox hazard model, sex remained a significant covariate in CD4 reconstitution in favor of females in the 1–4yr group (adjusted hazard ratio 1.4, p = 0.030) and also in the ≥5yo group (adjusted hazard ratio 1.4, p = 0.011) (Table 2). In the two age groups of children studied (1–4yo and ≥5yo) in whom the CD4 ART initiation treatment criteria were, respectively, a CD4% <25%, and an absolute CD4 count of <350 cells/ul, recovery to a CD4% of ≥35% and ≥750 cells/ul, respectively, was independently associated with female sex and with pre-ART CD4% and absolute CD4 count, respectively. Pre-ART viral load in either case was not associated with speed of immune reconstitution.

**Table 2:** Multivariate analysis of sex differences in CD4+ T cell recovery after initiating ART.

	Cox hazard model				
	n	HR^a^ (95% CI^b^)	p	aHR^c^ (95% CI)	p
**Age 1–4yrs, ART initiation guidelines CD4% <25%**					
**Sex**					
Male	253	Reference		Reference	
Female	197	1.4 (1.04–1.9)	0.027	1.4 (1.02–1.9)	0.030
**CD4%**					
<15%	296	Reference		Reference	
15–24%	154	1.5 (1.1–2.1)	0.011	1.5 (1.1–2.1)	0.012
**Viral load (log copies/ml)**					
≥6.0	136	Reference			
5.0–5.9	223	0.8 (0.5–1.1)	0.19		
<5.0	91	0.96 (0.6–1.5)	0.86		
**Age ≥5yrs, ART initiation guidelines CD4 <350/ul**					
**Sex**					
Male	225	Reference		Reference	
Female	186	1.5 (1.1–1.9)	0.007	1.4 (1.1–1.9)	0.011
**CD4 (counts/ul)**					
<200	241	Reference		Reference	
200–349	170	1.6 (1.2–2.1)	<0.0001	1.6 (1.2–2.1)	<0.001
**Viral load (log copies/ml)**					
≥6.0	226	Reference			
5.0–5.9	161	0.8 (0.4–1.4)	0.38		
<5.0	24	0.8 (0.4–1.4)	0.36		

^a^ HR: Hazard ratio

^b^ 95% confidential interval range

^c^ aHR: adjusted hazard ratio

### Higher frequency of female children initiating ART due to clinical disease

We next addressed the question of whether ART initiation in children occurred as a result of meeting clinical criteria (WHO clinical disease stage 3 or 4), as distinct from CD4 criteria, more frequently in females. Of the 222 children in whom ART was initiated for clinical indications in children whose CD4 counts were above CD4 treatment thresholds, 127 (57%) were female and 95 (43%) were male. This contrasts with the preponderance of male children (55%) starting ART as a result of CD4 criteria (p<0.001, Fig 2). This suggests that a higher frequency of clinical disease is suffered by female children who remain off ART as a result of CD4 counts being above the criteria for ART initiation.

To investigate further the reasons for ART initiation in children aged 1–4yo with CD4%>25% and in children aged ≥5yo with absolute CD4 counts of >350/ul, case records showed that clinical indications for ART initiation were specified in 166 of the 222 children (Table 3). TB disease was the clinical indication for ART initiation in 90 of these 166 cases, manifest as extrapulmonary TB, miliary TB, pulmonary TB, TB meningitis, or TB pericarditis. The sex difference, however, with respect to TB disease (47% were males, 53% females) was not statistically significant (p = 0.21, Fisher’s Exact test). The main sex difference in morbidity observed was in nutritional status, reflected in stunting, wasting, marasmus and kwashiorkor, which together represented the clinical indication for ART initiation in 38 children, of whom 30 were females (78%, p = 0.002, Fisher’s Exact test). In addition to the clinical indications for ART initiation there was a number of children in whom ART was initiated for reasons that were related to CD4 count and viral load but outside the guidelines (for example in children ≥5yo ART was initiated in 31 children in spite of absolute CD4 counts being >350 cells/ul, but because of low CD4%). However, it is clear that the sex difference in ART initiation in children with CD4 counts above the treatment threshold was largely due to higher instances of HIV disease in female children.

**Table 3:** Indications for ART Initiation in 222 children whose CD4 counts were above CD4 treatment thresholds.

	Male	Female	Total
Clinical indications:			
Abdominal/Extrapulmonary TB	5	8	13
Anal warts	1	0	1
Chronic lung disease, bronchiectasis	2	4	6
Cryptococcal meningitis	0	1	1
Herpes zoster	0	1	1
HIV Encephalopathy	4	2	6
Kaposi Sarcoma	1	1	2
Kwashiorkor	1	0	1
Lymphoid Interstitial Pneumonitis	5	5	10
Marasmic kwashiorkor +/- chronic diarrhoea	0	3	3
Miliary TB	0	1	1
Oral papillomata	0	1	1
*Pneumocystis carinii* pneumonia	1	1	1
Peripheral neuropathy	0	1	1
Pulmonary TB	35	37	72
Severe parotid enlargement	4	0	4
Stunting, severe stunting	3	6	9
TB Meningitis	1	2	3
TB Pericarditis	1	0	1
Thrombocytopenia	0	1	1
Varicella pneumonia	1	0	1
Wasting, severe wasting	5	21	26
Total	70	96	166
Non-clinical indications ^a^:			
Age ≥5yo CD4>350 but CD4%<25% ^a^	14	17	31
High viral load (>10^6^ copies/ml) ^a^	1	0	1
Indication for ART initiation not known:	10	14	24
Total	95	127	222

^a^ Not within the guidelines for ART initiation in children

## Discussion

Substantial differences between the sexes have been observed in children, both in incidence and outcome, from a range of infectious diseases [1, 2]. For reasons that remain largely unknown, female children and female adults alike appear to generate a more robust immune response to infections and vaccinations [6, 28]. The data presented here highlight several differences between male and female children following HIV infection that substantially pre-date the onset of adolescence.

In females, CD4 counts are higher both in HIV-infected children and even in HIV-uninfected newborn infants, as early as the first day of life. On average, female newborns have CD4 counts 266/ul higher than male newborns. Thus, the well-reported higher CD4 counts observed in female adults and older children in fact very likely pre-date birth and are independent of HIV infection. Not unexpectedly, therefore, in view of WHO CD4-based criteria for ART initiation, more male children received ART than females in the pediatric cohorts described here. However, immune reconstitution following ART initiation was faster and more complete (achieving normal CD4 counts for age) in female children, even after adjustment for baseline CD4 counts. Strikingly, although there was no sex difference in post-ART mortality, ART initiation for clinical reasons was significantly more likely in females, in contrast with ART being started as a result of meeting CD4 criteria mostly in males (p<0.001).

In the HIV-infected children studied here, the CD4 count differences (absolute counts and CD4%) that are apparent at birth persist throughout childhood into adolescence, with the same difference between males and females of approximately 100 cells/ul as reported in HIV-infected adults [18,19]. These pre-adolescence differences have been described in previous reports from North America and sub-Saharan Africa [20, 21]. Of note, the sex differences in CD4 counts and CD4% in HEU infants reported in one of these studies [21] are virtually identical to those described here. In both studies [20, 21], however, viral loads were somewhat lower in female children pre-ART, a finding that was not observed here, except in children aged ≥12yrs.

These findings have prompted the question of whether ART is initiated too late in female children as a result of adherence to CD4 criteria as the principal criterion for initiating ART [20–22]. In one study [21], there was a significantly higher post-ART mortality in female children, although virtually all of the patients in that cohort were receiving monotherapy or dual therapy with nucleoside analogues, and it is possible that the precise nature of the therapy might have affected outcome. In a more recent African study [22], female sex was an independent risk factor associated with increased mortality in ART-treated children. Here we noted no sex differences in mortality following ART initiation with standard first-line drug regimens. However ART initiation as a result of clinical criteria, in children whose CD4 counts were above the levels that would meet WHO criteria to start therapy, was observed in significantly more female than male children. Together these findings suggest that ART might reasonably be initiated at higher CD4 counts in female children in order to reduce the increased mortality [21, 22] and morbidity associated with ART initiation criteria that do not differentiate between the sexes.

The observation here that more males are receiving ART than females, and yet the numbers of females and males who are infected via mother-to-child transmission is equal [11] also suggests the possibility that several factors may be in operation. The first, as proposed above, is that females reach CD4 thresholds for ART initiation later than males. The second is that females progress more slowly to disease, for which there is no evidence. The third is that female children are less likely to be presented to clinic than male children; overall 49% of the 2,101 HIV-infected children attending Kimberley clinic were females.

In terms of immune reconstitution post-ART, normal CD4 counts for uninfected age-matched children were achieved more rapidly by, and in greater numbers of, female children than males, independent of pre-ART CD4 counts. These findings contrast somewhat to the more rapid decline in CD4% in female children during structured treatment interruption [29] but mirror those in adults [30, 31], showing greater rises in CD4 counts after ART initiation in women, even after adjustment for baseline CD4 counts; and are consistent also with similar observations of more rapid immune reconstitution in older prepubertal female children in a Thai study [32].

The proposed mechanism that oestrogen and other hormones [18] underlie sex differences in CD4 counts may initially appear unlikely, given that differential CD4 counts between sexes arise at birth. However, changes in sex hormones are not limited to puberty; in fact dramatic hormonal changes are seen during the ‘minipuberty’ in the first 6 months after birth [33–35]. Oestrogen appears to play a central role in CD4 T cell development and function [36], while testosterone has a broadly immunosuppressive role [37]. The combination of immune responses being modulated by sex hormones [37], of dosage differences in X-linked genes, and of genes on autosomal chromosomes with sex-biased expression, may all contribute to these observed sex differences.

One potential mechanism suggested to underlie sex differences in adult HIV infection is immune activation. The higher levels of IFN-α produced by plasmacytoid dendritic cells of women compared to men in response to HIV-1 encoded TLR-7 ligands have been proposed to explain the faster HIV progression rates in women for a given viral load or CD4 count [38]. However, no sex difference in immune activation was observed in a previous pediatric study in South African HIV-infected children [20].

The sex differences in CD4 count, VL and immune reconstitution post-ART are not associated with major differences in disease outcome in pediatric HIV infection. Several studies have shown a higher in utero HIV infection rate in females [39–43]. This would suggest either that females are more susceptible to in utero infection, or alternatively that males have more rapid disease progression and die in utero. It is noteworthy that, while congenital CMV infection rates are similar in males and females, severe congenital CMV disease is twice as likely in females [44]. Cerebral damage in congenitally CMV-infected fetuses is correlated with the presence of infiltrating activated cytotoxic CD8+ T cells in infected brain tissue, consistent with a more vigorous immune response in female fetuses contributing to immunopathology [45, 46].

One further discussion point raised by these data is the pragmatic consideration that, lack of access to timely CD4 testing and increasing benefit-to-risk ratio of ART together may argue in favor of initiating treatment for all HIV-infected children and adolescents, irrespective of age, CD4 count or clinical indications. This strategy has recently been initiated in some countries such as Uganda, and would have the advantage of ensuring that females do not experience greater morbidity as a result of ART initiation based on inadequately understood thresholds.

In conclusion, this analysis of the influence of sex differences on outcome in HIV-infected children in South Africa indicates that females have higher CD4 counts from birth in infected and uninfected children, and improved immune reconstitution on ART compared to males following ART initiation. As a result, ART initiation is more likely in males based on CD4 criteria, and in females based on clinical disease progression, even when CD4 counts are above the WHO threshold for ART initiation. Although there was no evidence that these sex differences in CD4 counts resulted in significantly increased mortality in female children as a result of ART being initiated too late in females, the increased morbidity suffered by female compared with male children suggests ART is initiated later than optimal in females. Future studies should evaluate morbidity, growth and clinical outcomes of females compared to males following ART initiation.

## Supporting Information

S1 Table Data sheets used for analysis. (XLSX)

S2 Table Lack of differences among male and female children in pre-ART absolute CD4+ T cell count, CD4% and viral load in children prior to initiating ART. (XLSX)
